# Results from the national sepsis practice survey: predictions about mortality and morbidity and recommendations for limitation of care orders

**DOI:** 10.1186/cc7926

**Published:** 2009-06-23

**Authors:** James M O'Brien, Scott K Aberegg, Naeem A Ali, Gregory B Diette, Stanley Lemeshow

**Affiliations:** 1 Division of Pulmonary, Allergy, Critical Care and Sleep Medicine, Center for Critical Care, Department of Internal Medicine, The Ohio State University Medical Center, 201 Davis HLRI, 473 West 12thAvenue, Columbus, OH 43210, USA; 2 Division of Pulmonary and Critical Care Medicine, Department of Internal Medicine, Johns Hopkins School of Medicine, 1830 East Monument, 5th Floor, Baltimore, MD 21205, USA; 3 College of Public Health, The Ohio State University, 320 West 10thAvenue, M-116 Starling-Loving Hall, Columbus, OH 43210, USA

## Abstract

**Introduction:**

Critically ill patients and families rely upon physicians to provide estimates of prognosis and recommendations for care. Little is known about patient and clinician factors which influence these predictions. The association between these predictions and recommendations for continued aggressive care is also understudied.

**Methods:**

We administered a mail-based survey with simulated clinical vignettes to a random sample of the Critical Care Assembly of the American Thoracic Society. Vignettes represented a patient with septic shock with multi-organ failure with identical APACHE II scores and sepsis-associated organ failures. Vignettes varied by age (50 or 70 years old), body mass index (BMI) (normal or obese) and co-morbidities (none or recently diagnosed stage IIA lung cancer). All subjects received the vignettes with the highest and lowest mortality predictions from pilot testing and two additional, randomly selected vignettes. Respondents estimated outcomes and selected care for each hypothetical patient.

**Results:**

Despite identical severity of illness, the range of estimates for hospital mortality (5^th ^to 95^th ^percentile range, 17% to 78%) and for problems with self-care (5^th ^to 95^th ^percentile range, 2% to 74%) was wide. Similar variation was observed when clinical factors (age, BMI, and co-morbidities) were identical. Estimates of hospital mortality and problems with self-care among survivors were significantly higher in vignettes with obese BMIs (4.3% and 5.3% higher, respectively), older age (8.2% and 11.6% higher, respectively), and cancer diagnosis (5.9% and 6.9% higher, respectively). Higher estimates of mortality (adjusted odds ratio 1.29 per 10% increase in predicted mortality), perceived problems with self-care (adjusted odds ratio 1.26 per 10% increase in predicted problems with self-care), and early-stage lung cancer (adjusted odds ratio 5.82) were independently associated with recommendations to limit care.

**Conclusions:**

The studied clinical factors were consistently associated with poorer outcome predictions but did not explain the variation in prognoses offered by experienced physicians. These observations raise concern that provided information and the resulting decisions about continued aggressive care may be influenced by individual physician perception. To provide more reliable and accurate estimates of outcomes, tools are needed which incorporate patient characteristics and preferences with physician predictions and practices.

## Introduction

Sepsis affects at least 750,000 patients annually in the USA with incidence increasing at a rate of approximately 1.5% per year [1,2]. Critically ill patients, including those with sepsis, and their families desire prognostic information early in the hospital course to help inform decisions about continued supportive care, even when such information is uncertain [3]. Such early provision of prognostic information and shared decision-making, including clinician recommendations about appropriate treatments and goals of care, are evidence-based endorsements of the American College of Critical Care [4] and the Surviving Sepsis Campaign [5]. However, the patient and provider factors that influence physician prognostication in the intensive care unit (ICU) are largely unknown.

A series of reports from the Level of Care Study suggest that such physician predictions are influential on subsequent care and outcome. Based on their observations, physician predictions about ICU mortality and recovery are strongly predictive of subsequent withdrawal of mechanical ventilation [6], do-not-resuscitate (DNR) orders [7], and ICU mortality [8]. Therefore, better understanding of the factors that influence physician prognostication may allow for an appreciation for the mechanisms underlying factors associated with poorer outcomes among septic patients and improved risk-adjusting methodology, which could incorporate physician intuition with clinical data.

In a national survey of physicians with experience treating sepsis, we used simulated clinical vignettes to measure physician predictions about outcomes from septic shock, to test the influence of selected patient factors on these predictions and to determine how these factors and predictions affect recommendations for limitation of care. We hypothesized that physician estimates of outcomes would vary widely. We also believed that patient factors obvious to a treating clinician (older age, body mass index (BMI) for obesity, and cancer diagnosis) would be associated with higher estimates of mortality, despite identical measures of acute illness severity. Finally, we hypothesized that increasing estimates of mortality and morbidity and clinical factors would be associated with suggestions for limitations of care when no patient preference was provided.

## Materials and methods

### Study sample and administration

We randomly selected potential subjects from members of the Critical Care Assembly of the American Thoracic Society with a US mailing address. The study was reviewed by the Planning Committee of the Assembly and approved by the Ohio State University Biomedical Institutional Review Board. From 18 June to 24 September, 2007, we mailed self-administered surveys including a letter explaining the study purpose and a stamped return envelope. The initial mailing included $10 cash incentive. Non-respondents received a duplicate survey 30 days after the initial mailing with no additional incentive. Surveys returned for inaccurate addresses and by those who do not care for septic adults were replaced by random selection.

### Questionnaire

We developed study vignettes through focus groups and a pilot administration to intensivists at The Ohio State University Medical Center. Vignettes involved a male patient with community-acquired pneumonia who received initial care, including mechanical ventilation, volume resuscitation, and antibiotics. All had an acute physiology and chronic health evaluation (APACHE) II score of 25 with sepsis-associated shock, respiratory failure, and lactic acidosis. The patient was admitted to the ICU for further care. No patient preferences regarding goals of care were provided.

Each vignette had either a normal BMI (22 kg/m^2^) or an obese BMI (40 kg/m^2^), was either younger (50 years) or older (70 years), and had either no co-morbidities or recently diagnosed stage IIA non-small cell lung cancer. Obesity was of interest because of our prior work [9,10] and because it is consistently associated with negative physician attitudes [11,12] but is not consistently associated with outcomes [13,14]. We studied age to extend observations about aggressiveness of care in elderly patients with serious illnesses [15] and to determine the effect age has on physician decision-making beyond its contribution to APACHE II score. We included a recent diagnosis of a potentially curable cancer [16] to evaluate the effect of a chronic condition on predictions about acute illness. All respondents received the vignettes with the lowest [see Additional data file 1] (50 years old, no co-morbidities, normal BMI) and highest (70 years old, stage IIA non-small cell lung cancer, obese BMI) mortality rates in pilot testing. Two additional vignettes were randomly selected for each survey with weighting designed to provide adequate sample sizes for comparisons of interest. The order of the vignettes within each survey was random.

For each vignette, the respondent was asked if he or she would choose additional therapies, and, if so, which ones. Respondents were asked to predict outcomes, including the probability of hospital survival without additional interventions chosen after ICU admission (referred to as 'baseline mortality') and the probability of the patient being able to wash and dress himself six months after hospital discharge (assuming survival). Respondents indicated their prediction by placing an 'X' on visual-analog scale, represented by a 10 cm horizontal line. All outcome predictions were determined by measuring the location of the X placed on the visual-analog scale in mm. We also collected demographic information about respondents.

### Sample size and statistical plan

Our primary hypothesis was that the studied patient factors would be associated with the predicted probability of hospital survival without additional interventions chosen after ICU admission (or baseline mortality). Our secondary hypotheses were that between-respondent estimates would have a wide range despite identical patient factors and that mortality and morbidity predictions and vignette factors were associated with recommendations to limit care. We classified choices of a DNR order, restriction of further escalation of care, and/or termination of supportive care as recommendations to limit care.

We used data from pilot testing for sample size calculations. We planned to demonstrate at least a 10% difference in baseline mortality between pairs of vignettes of interest with a two-sided alpha of 0.05 and power of 0.8 and expected a 50% response rate. This required an estimated sample of 355 completed surveys. We used the 5th to the 95th percentile of the estimated mortality predictions (inclusive of 90% of respondents) for each vignette as a measure of the variability in these predictions.

The unit of analysis for all results was the individual study vignette. Each respondent completed multiple vignettes (up to four), so we used analyses which accounted for this non-independence. We considered responses to the same vignette by different respondents to be independent. All tables display the association in such analyses including either a single independent variable ('univariable') or multiple independent variables ('multivariable') in linear or logistic regression models, as appropriate.

For the final risk-adjusting analyses with physician predictions as the outcome variable, we included the clinical factors from the vignettes (regardless of statistical significance) and studied respondent factors, which were significantly associated with the prediction (*P *< 0.05) and/or altered the parameter estimate or odds ratio of any of the patient factors by at least 15%. For the risk-adjusting analyses for recommendation to limit care with curative intent, we included the clinical factors from the vignettes (regardless of statistical significance), the prediction about baseline mortality, and problems with washing and dressing oneself in six months, assuming survival (regardless of statistical significance). We also included respondent factors which were significantly associated with the recommendation to limit care (*P *< 0.05) and/or altered the parameter estimate or odds ratio of any of the patient factors or predictions by at least 15%. We analyzed continuous variables with fractional polynomials to determine if transformation or categorization was appropriate and in no instance was this suggested. We used SAS (v9.1, SAS Institute, Inc., Cary, NC, USA) or STATA (SE10.0, StatCorp LP, College Station, TX, USA) for all analyses. These data were previously presented in abstract form at the 2008 American Thoracic Society International Conference.

## Results

### Respondents

**Figure 1:**
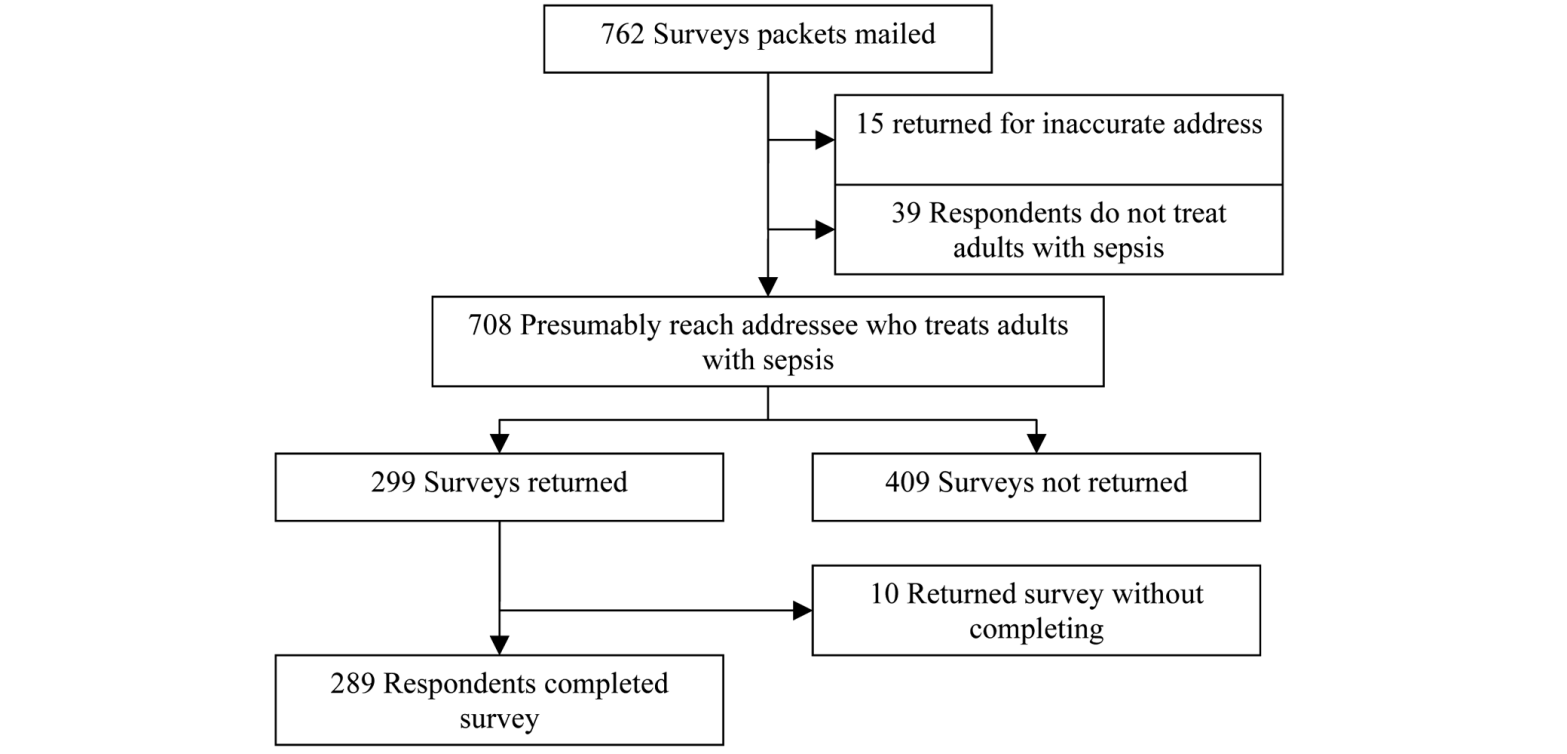
Responses to National Sepsis Practice Survey.

After both mailings, we received a response rate of 40.8%, representing 81.4% of the projected sample size (Figure 1). Nearly all respondents (99%) reported caring for at least one septic patient per week and most had moderate or extensive self-rated experience in treating sepsis (Table 1). Among the completed vignettes with normal or obese BMIs, with younger and older ages, and with no co-morbidities and early-stage lung cancer, there were no statistically significant differences in respondent characteristics (data not shown).

**Table 1:** Respondent demographic and practice characteristics

Age, years, mean (SD)	45.7 (9.5)
Years since medical school graduation, mean (SD)	19.9 (9.7)
Decade of medical school graduation, number (%)	
1960s	9 (3.1%)
1970s	65 (22.5%)
1980s	87 (30.1%)
1990s	104 (36.0%)
2000s	24 (8.3%)
Primary employer, number (%)	
Private practice, community hospital, and/or managed care	160 (55.6%)
Academic medical center and/or University medical school	108 (37.5%)
Other	20 (6.9%)
Weight, pounds, mean (SD)	173.6 (30.6)
Height, inches, mean (SD)	68.7 (4.3)
BMI, kg/m^2^, mean (SD)	26.0 (4.3)
Underweight BMI (<18.5 kg/m^2^), number (%)	2 (0.7%)
Normal BMI (18.5 to 24.9 kg/m^2^), number (%)	126 (43.9%)
Overweight BMI (25 to 29.9 kg/m^2^), number (%)	124 (43.2%)
Obese BMI (=30 kg/m^2^), number (%)	35 (12.2%)
Estimates of BMI of respondent's ICU patients, mean (SD)	
Underweight BMI (<18.5 kg/m^2^)	10.4% (6.5)
Normal BMI (18.5 to 24.9 kg/m^2^)	26.8% (13.5)
Overweight BMI (25 to 29.9 kg/m^2^)	30.6% (11.7)
Obese BMI (30 to 39.9 kg/m^2^)	22.8% (11.7)
Severely obese BMI (=40 kg/m^2^)	9.4% (6.5)
Self-reported chronic health problem, number (%)	45 (16.2%)
Percentage of job spent in direct care of ICU patients, mean (SD)	38.8% (22.2)
Number of septic patients cared for per week, number (%)	
0	3 (1.0%)
1 to 2	62 (21.7%)
3 to 5	115 (40.2%)
6 to 10	71 (24.8%)
11 to 15	24 (8.4%)
15+	11 (3.8%)
Self-rated experience treating sepsis, number (%)	
Limited	1 (0.4%)
Moderate	109 (38.0%)
Extensive	177 (61.7%)
Specialty, number (%)	
Internal medicine	154 (54.0%)
Family medicine	1 (0.4%)
Surgery	3 (1.0%)
Anesthesia	3 (1.0%)
Pulmonary	267 (93.0%)
Critical care	262 (91.3%)
Sleep medicine	66 (23.0%)
Other	13 (4.5%)

BMI = body mass index; ICU = intensive care unit; SD = standard deviation.

### Predicted probability of baseline hospital mortality

For all patients described in the vignettes, the median baseline mortality (the predicted hospital mortality if no additional therapies were added after ICU admission) was 47% (range from 5th to 95th percentile 17% to 78%). When grouped by vignette, the ranges of mortality estimates remained wide (Table 2). For each respondent, the average difference between the highest and lowest baseline mortality prediction was 24.9 percentage points (95% confidence interval (CI) 23.2 to 26.7 percentage points). Despite identical APACHE II scores and organ failure, older age, early-stage lung cancer, and an obese BMI were all associated with higher predictions of baseline mortality (Table 3). No measured respondent factors were associated with the baseline mortality prediction.

**Table 2:** Predicted hospital mortality, based on clinical factors in study vignettes

Vignette characteristics					Predicted 'baseline' hospital mortality	
BMI	Age (years)	Co-morbidities	APACHE II Score	Number of vignettes	Median	5^th ^percentile to 95^th ^percentile range

Normal	50	None	25	285	37%	10% to 69%
Obese	50	None	25	133	42%	16% to 74%
Normal	50	Early-stage lung cancer	25	43	44%	21% to 72%
Normal	70	None	25	123	46%	15% to 74%
Obese	50	Early-stage lung cancer	25	42	49%	24% to 80%
Obese	70	None	25	130	50%	28% to 80%
Normal	70	Early-stage lung cancer	25	98	53.5%	20% to 84%
Obese	70	Early-stage lung cancer	25	287	55%	27% to 81%
All vignettes				1141	47%	17% to 78%

Respondents were asked to provide estimates of hospital mortality (see *Methods *for details). The median and central 90% of responses are shown based on vignette characteristics.

APACHE II = acute physiology, age and chronic health evaluation II; BMI = body mass index.

**Table 3:** Patient factors in vignettes and predicted 'baseline' mortality

	Univariable analyses		Multivariable analyses	
	Percentage point increase in predicted mortality (95% confidence interval)	*P *value	Percentage point increase in predicted mortality (95% confidence interval)	*P *value

70 years old (versus 50 years old)	12.1 (10.0 to 14.2)	<0.0001	8.2 (6.1 to 10.4)	<0.0001
Stage IIA NSCLC (versus no cancer)	10.8 (8.7 to 13.0)	<0.0001	5.9 (3.6 to 8.1)	<0.0001
BMI 40 kg/m^2^ (versus 22 kg/m^2^)	8.6 (6.4 to 10.7)	<0.0001	4.3 (2.5 to 6.2)	<0.0001

Baseline mortality was considered the predicted mortality if no additional care, other than the care instituted prior to admission to the intensive care unit (ICU), was added. The univariable estimates include only the variable indicated in the model while multivariable estimates included all variables with displayed estimates in that column. All analyses accounted for non-independence of responses due to respondents completing multiple vignettes.

BMI = body mass index; NSCLC = non-small cell lung carcinoma.

### Predicted probability of problems with self-care among survivors

For all patients described in the vignettes, the median predicted rate of problems among survivors with washing and dressing oneself was 25% (range from 5th to 95th percentile 2% to 74%). As with the baseline mortality predictions, among vignettes with identical patient factors, these ranges of predictions were wide (Table 4). Older age, early-stage lung cancer, and an obese BMI were all associated with higher probabilities of problems with self-care at six months among survivors (Table 5). After adjustment for the clinical factors in the vignettes, respondents who were older and reported chronic health problems predicted fewer problems with self-care for surviving patients than respondents who were younger and who had no health problems (Table 6). After adjustment for these respondent factors, higher BMI, older age, and a cancer diagnosis continued to be associated with higher predicted difficulties with self-care among survivors.

**Table 4:** Predicted problems with self-care, based on clinical factors in study vignettes

Vignette characteristics					Predicted problems washing and dressing self at six months (assuming survival)	
BMI	Age (years)	Co-morbidities	APACHE II Score	Number of vignettes	Median	5^th ^percentile to 95^th ^percentile range
Normal	50	None	25	285	12%	1% to 58%
Obese	50	None	25	133	20%	3% to 66%
Normal	50	Early-stage lung cancer	25	43	25%	3% to 55%
Normal	70	None	25	124	25%	4% to 69%
Obese	50	Early-stage lung cancer	25	42	28.5%	2% to 70%
Obese	70	None	25	130	32%	4% to 74%
Normal	70	Early-stage lung cancer	25	98	31.5%	4% to 77%
Obese	70	Early-stage lung cancer	25	287	39%	8% to 81%
All vignettes				1142	25%	2% to 74%

Respondents were asked to provide estimates of problems washing and dressing himself at six months, assuming the patient survived (see *Methods *for details). The median and central 90% of responses are shown based on vignette characteristics. APACHE II = acute physiology, age and chronic health evaluation II; BMI = body mass index.

**Table 5:** Patient factors in vignettes and predicted problems with self-care, univariable analyses

	Univariable analyses	
	Percentage point increase in predicted problems with self-care in six months (95% confidence interval)	*P *value

70 years old (versus 50 years old)	16.1 (14.0 to 18.1)	<0.0001
Stage IIA NSCLC (versus no cancer)	13.5 (11.3 to 15.7)	<0.0001
BMI 40 kg/m^2^ (versus 22 kg/m^2^)	10.9 (9.2 to 12.6)	<0.0001
Respondent age (per decade of age)	-4.5 (-6.5 to -2.5)	<0.0001
Respondent self-reported chronic health condition	-7.3 (-11.8 to -2.8)	0.0016

Respondents were asked to predict the probability of each patient having difficulties with washing and dressing himself in six months, assuming the patient survived. Univariable estimates include only the variable indicated in the model. Analyses accounted for non-independence of responses due to respondents completing multiple vignettes.

BMI = body mass index; NSCLC = non-small cell lung carcinoma.

**Table 6:** Patient factors in vignettes and predicted problems with self-care, multivariable analyses

	Multivariable analysis		Multivariable analysis, including respondent factors	
	Percentage point increase in predicted problems with self-care in six months (95% confidence interval)	*P *value	Percentage point change in predicted problems with self-care in six months (95% confidence interval)	*P *value

70 years old (versus 50 years old)	11.5 (9.0 to 13.9)	<0.0001	11.6 (9.2 to 13.9)	<0.0001
Stage IIA NSCLC (versus no cancer)	6.8 (4.3 to 9.3)	<0.0001	6.9 (4.3 to 9.4)	<0.0001
BMI 40 kg/m^2^ (versus 22 kg/m^2^)	5.4 (3.3 to 7.5)	<0.0001	5.3 (3.2 to 7.4)	<0.0001
Respondent age (per decade of age)			-4.0 (-6.0 to -2.0)	0.0001
Respondent self-reported chronic health condition			-5.8 (-10.1 to -1.4)	0.0103

Respondents were asked to predict the probability of each patient having difficulties with washing and dressing himself in six months, assuming the patient survived. Multivariable estimates included all variables with displayed estimates in that column. All analyses accounted for non-independence of responses due to respondents completing multiple vignettes.

BMI = body mass index; NSCLC = non-small cell lung carcinoma.

### Recommendations to limit care with curative intent

Limitation of care with curative intent was suggested in 9.1% of vignettes. Most commonly, a DNR order alone (78.4% of those with limitation recommendation) was suggested. In univariable analyses, early-stage lung cancer, older age, an obese BMI and predictions of increased baseline mortality and problems with self-care were associated with limitations of care suggestions (Table 7). In multivariable analyses accounting for other vignette factors, an obese BMI was not associated with limitation of care (Table 8). Once adjusted for predictions about mortality and problems with self-care, older age was also not associated with suggestions to limit care. In the final multivariable model, every 10% increase in predicted baseline mortality and in predicted problems with self-care was independently associated with 29% and 26% increased odds of limitation of care, respectively. A cancer diagnosis was associated with nearly six-fold increased odds of limitation of care in the final multivariable model. In other words, respondents were significantly more likely to recommend limitations in aggressive care for a patient with early-stage lung cancer compared with one without cancer, even when the vignettes had identical mortality and morbidity predictions. Respondents with BMIs suggesting overweight or obesity were significantly less likely to suggest a limitation of care order.

**Table 7:** Factors associated with suggested limitation of care orders, univariable analysis

	Univariable analyses	
	Odds ratio (95% CI)	*P *value

70 years old (versus 50 years old)	6.76 (3.96 to 11.55)	<0.0001
Stage IIA NSCLC (versus no cancer)	10.95 (6.30 to 19.03)	<0.0001
BMI 40 kg/m^2^ (versus 22 kg/m^2^)	2.54 (1.78 to 3.62)	<0.0001
Baseline predicted hospital mortality (per 10% increase)	1.63 (1.41 to 1.89)	<0.0001
Predicted problems with self-care at six months (per 10% increase)	1.46 (1.32 to 1.62)	<0.0001
Overweight or obese respondent BMI	0.55 (0.33 to 0.93)	0.0258

Limitations of care included suggesting a 'do not resuscitate' order, that there be no further escalation of care (e.g., no addition of vasopressors), and/or termination of supportive care with appropriate 'comfort care' measures. Mortality predictions were estimated prior to any additional chosen care, including limitation of care orders. Respondents were asked to predict the ability to perform self-care (wash and dress oneself) in six months, assuming survival. Univariable estimates include only the variable indicated in the model. Analyses accounted for non-independence of responses due to respondents completing multiple vignettes.

BMI = body mass index; CI = confidence interval; NSCLC = non-small cell lung carcinoma.

**Table 8:** Factors associated with suggested limitation of care orders, multivariable analyses

	Multivariable analyses					
	Adjusted odds ratio (95% CI)	*P *value	Adjusted odds ratio (95% CI)	*P *value	Adjusted odds ratio (95% CI)	*P *value

70 years old (versus 50 years old)	2.90 (1.55 – 5.44)	0.0009	1.90 (0.98 to 3.68)	0.0589	1.87 (0.95 to 3.66)	0.0685
Stage IIA NSCLC (versus no cancer)	7.12 (3.75 – 13.52)	<0.0001	5.70 (2.97 to 10.93)	<0.0001	5.84 (3.05 to 11.20)	<0.0001
BMI 40 kg/m^2^ (versus 22 kg/m^2^)	1.18 (0.78 – 1.79)	0.4274	1.02 (0.66 to 1.57)	0.9375	1.01 (0.65 to 1.56)	0.9694
Baseline predicted hospital mortality (per 10% increase)			1.30 (1.10 to 1.54)	0.0019	1.29 (1.09 to 1.53)	0.0027
Predicted problems with self-care at six months (per 10% increase)			1.26 (1.12 to 1.41)	<0.0001	1.26 (1.12 to 1.42)	0.0002
Overweight or obese respondent BMI					0.53 (0.29 to 0.96)	0.0345

Limitations of care included suggesting a 'do not resuscitate' order, that there be no further escalation of care (e.g., no addition of vasopressors), and/or termination of supportive care with appropriate 'comfort care' measures. Mortality predictions were estimated prior to any additional chosen care, including limitation of care orders. Respondents were asked to predict the ability to perform self-care (wash and dress oneself) in six months, assuming survival. Multivariable estimates included all variables with displayed estimates in that column. All analyses accounted for non-independence of responses due to respondents completing multiple vignettes.

BMI = body mass index; CI = confidence interval; NSCLC = non-small cell lung carcinoma.

Because of the generally poor outcome for septic patients requiring cardiopulmonary resuscitation (21), some respondents might not consider a DNR order as a change in the goals of care. We recalculated our analyses considering only limitations of supportive care that included a non-escalation order and/or a change to comfort care (n = 22, 1.96% of vignettes). The results of these analyses were very similar in magnitude and direction to those including DNR as a limitation of care with curative intent (data not shown), although respondent BMI was no longer associated with the limitation of care.

## Discussion

In this mail-based survey of physicians with experience caring for septic patients, physician predictions about hospital mortality in septic shock varied widely, even when clinical information was identical. Beyond this variability, older age, an obese BMI, and cancer diagnosis were associated with predictions for greater mortality and morbidity. These findings suggest that physicians incorporate clinical factors into their estimates, which are independent of validated severity of illness scores. These prognostic estimates and the hypothetical patient's diagnosis of early-stage lung cancer were also associated with recommendations to limit care.

Severity of illness scoring systems were developed in an attempt to objectively quantify the risk of hospital mortality to 'evaluate the outcomes of care' [17]. These systems, however, were not designed for prognostication of individual patients [18]. They also may have less ability to discriminate between survivors and nonsurvivors than ICU physicians, although discriminatory capacity is only moderate among ICU physicians [19]. Despite these limitations, physicians are advised to provide prognostic information and recommendations about appropriate treatments and goals of care by the American College of Critical Care [4] and the Surviving Sepsis Campaign [5]. These predictions are then influential on subsequent care and outcome [6-8]. The patient and provider factors that color the information provided by ICU physicians are largely unknown. By better understanding these factors, it may allow for the development of interventions that should be directed at the patient's illness and ones which should be directed at providing more accurate tools for discriminating outcomes for individual patients. Differences in provider tendencies in prognostication and communication with patients and families could affect the results of observational studies as well. Although adjusting for differences in the clinical status of patients is common, most studies do not incorporate physician predictions or even patient preferences about continued life support in studies of risk factors for outcomes from critical illness.

When presented with identical clinical data, individual physicians experienced in treating sepsis made dramatically different estimates of mortality. The narrowest range (5th to 95th percentile of values) of predictions across respondents was 51 percentage points. In other words, one would not be surprised if two physicians, presented with the same information, would provide estimates of mortality that differed by more than 50 percentage points. Such prognostic variation and disagreement have been reported previously [20] and could influence the expectations of recovery each physician communicates to patients and families. Although we collected limited information about respondents, no measured factor appeared to consistently explain why a respondent might be more optimistic or pessimistic about hospital survival. Older respondents and those with a chronic health condition had more optimistic predictions about the ability of survivors to be independent at six months. This observation raises the possibility that a physician's expectations of recovery are influenced by his or her own health status. Further study should evaluate respondent factors that drive physician predictions and that affect subsequent decisions about continued aggressive care.

Despite identical acute severity of illness measures, respondents predicted poorer short-term outcomes for patients with high BMIs, older age, or limited-stage lung cancer. These findings suggest that physicians use information beyond that contained in severity of illness systems to generate estimates of proximate outcomes for septic shock patients. As physician prognostication may be equivalent or superior to that supplied by severity of illness systems [19], inclusion of these clinical factors may be appropriate. However, their potential prognostic relevance does not provide rationale for the observed variability in predictions.

Beyond provided prognostic information, recommendations regarding the value of continued aggressive care may influence ultimate outcome and not merely hasten the time to certain death. Those with limitation of care orders have higher risk-adjusted mortality for at least one year after ICU admission [21]. We found that poorer expected prognoses were associated with greater odds of recommending a limitation of care with curative intent. Older age and early-stage lung cancer were also associated with higher odds of a suggestion to limit care with curative intent. In the case of the older vignettes, this was mediated by expectations of poorer outcomes. However, even after considering its higher associated estimates of mortality and morbidity, early-stage lung cancer was associated with nearly six-fold increased odds of limitation of care suggestions. Although this may be partly explained by other outcome predictions unmeasured in this study (e.g., increased longer-term mortality among those surviving sepsis), the magnitude of this association is consistent with a higher perceived mortality for lung cancer patients than is supported by existing data [22-24]. We do not imply that the observation of higher rates of suggestions to limit care necessarily represents an inappropriate recommendation. Some studies suggest that general severity of illness systems (such as APACHE II) perform poorly for cancer patients in the ICU and may be overly optimistic, compared with systems developed specifically for ICU patients with cancer [25]. However, we suspect that if respondents were influenced by such inaccuracies for cancer patients, the association between recommendations to limit care and cancer diagnosis would be mediated by higher estimates of mortality, rather than being independent of these predictions.

There are important limitations to our study which limit its applicability to actual clinical practice and communications with families. Case-based vignettes are a simulated clinical situation and may not reflect predictions made about real patients. However, vignette-based studies have been found to be a valid measure of delivered care [26,27]. We forced respondents to provide prognostic information early in the clinical course. Although it is possible that early predictions lose relevance, one study suggests that events 48 hours after ICU admission have little effect on mortality predictions, compared with those made at ICU admission [28]. Also, the majority of surrogate decision-makers seek prognostic information early in a patient's illness, even in the face of uncertainty [3], making these early predictions more relevant. We also used a visual-analogue scale to measure respondent predictions. Although this method has been used for many studies and is an element of validated tools, such as the EuroQol-5D, it has not been specifically validated for physician predictions about septic patient vignettes.

We did not allow respondents to comment on the confidence each had in his or her predictions. Such questions would have allowed us to determine if a respondent felt confident enough to make a prediction about ultimate outcome and if he felt the estimates by another respondent were likely or not. A prior vignette-based study found that confidence in recommendations about care (ranging from 'comfort only' to 'full aggressive care') was higher among intensivists that nurses or residents and among respondents choosing care at one of the two extremes [26]. However, considerable disagreement between respondents remained even when respondents were highly confident. We also did not measure estimates of longer-term mortality, which some might argue is more relevant to decisions about continued ICU care. However, proximate measures of survival, including hospital mortality, have been accepted measures of efficacy of therapies in critically ill patients [29,30]. Our results also suggest that even such short-term prognostic estimates are associated with recommendations to limit care with curative intent.

Generalizability of our findings beyond those forming the study cohort is unknown, especially for clinicians who do not practice in the USA, those who do not regularly care for ICU patients, non-medical intensivists, or non-physician providers. We cannot comment on the potential influences of patient factors other than those controlled for in the vignettes on physician predictions and decision-making. A BMI of 40 kg/m^2 ^may be less compelling when written as part of a case than when it is observed in an ICU and, thus, we may have underestimated the influence that patient obesity has on physician predictions. We also cannot comment on the influence of unstudied respondent factors, such as ethnicity and religious affiliation, which might affect recommendations to limit aggressive care [31]. Our response rate was below our projections, but it exceeded the reported rates of many mail-based survey studies involving physicians [32,33]. By incorporating randomization, a non-responder was as likely to receive a vignette as a responder, reducing the likelihood of biased results.

## Conclusions

Given the wide range in predictions about mortality and morbidity and their association with recommendations for limitation of care, future research should focus on the patient and provider factors that produce such disparate predictions about outcomes. This is of particular importance in situations in which variation in predictions is associated with subsequent differences in provided care. For example, better tools to aid physician prognostication could reduce variation in such estimates and result in more uniform recommendations about continued aggressive care. Although severity of illness systems are attempts to provide such consistency, they ignore the additional information incorporated by a bedside clinician. Additional study is needed to better understand these subtleties that consistently (and inconsistently) influence physician predictions and practices. Without attending to the role of the provider in patient outcomes, we ignore aspects of the therapeutic relationship which may be more easily modified than patient characteristics and the severity of his/her acute illness.

## Key messages

• Among physicians experienced in caring for patients with septic shock, predictions about mortality and morbidity vary widely.

• Older age, high BMI, and early-stage lung cancer are associated with poorer predictions of mortality and morbidity, independent of acute severity of illness.

• Poorer outcome predictions are associated with an increased likelihood of a clinician suggesting a limitation of care order.

• Early-stage lung cancer is associated with higher odds of a suggestion of a limitation of care order, independent of predictions about mortality and morbidity.

## Abbreviations

APACHE II: acute physiology, age and chronic health evaluation II; BMI: body mass index; CI: confidence interval; DNR: do not resuscitate; ICU: intensive care unit.

## Competing interests

The authors declare that they have no competing interests.

## Authors' contributions

JMO conducted the pilot studies, designed the final survey, compiled the results, conducted the analyses, and drafted the manuscript. SKA participated in the design of the survey and helped to draft the manuscript. NAA participated in the design of the survey and helped to draft the manuscript. GBD participated in the design of the survey and helped to draft the manuscript. SL participated in the design of the survey, assisted with the analyses and helped to draft the manuscript. All authors approved the final draft of the manuscript.

## Supplementary Material

Additional file 1 Additional data file 1 is a JPG file containing a figure showing the 'lowest risk' vignette. The shaded areas indicate where there were variations between the vignettes (e.g. 50 years old vs. 70 years old). Each respondent received four vignettes. The first two were constant for all respondents and included this 'lowest risk' vignette and the 'highest risk' vignette (70 years old, stage IIA non-small cell lung cancer, and obese body mass index). The remaining two vignettes were randomly selected. Click here for file
