# Supplementary figures and images for: Results from the national sepsis practice survey: predictions about mortality and morbidity and recommendations for limitation of care orders
Source: Crit Care. 2009 Jun 23;13(3):R96. doi: 10.1186/cc7926 (PMC2717468; doi:10.1186/cc7926)

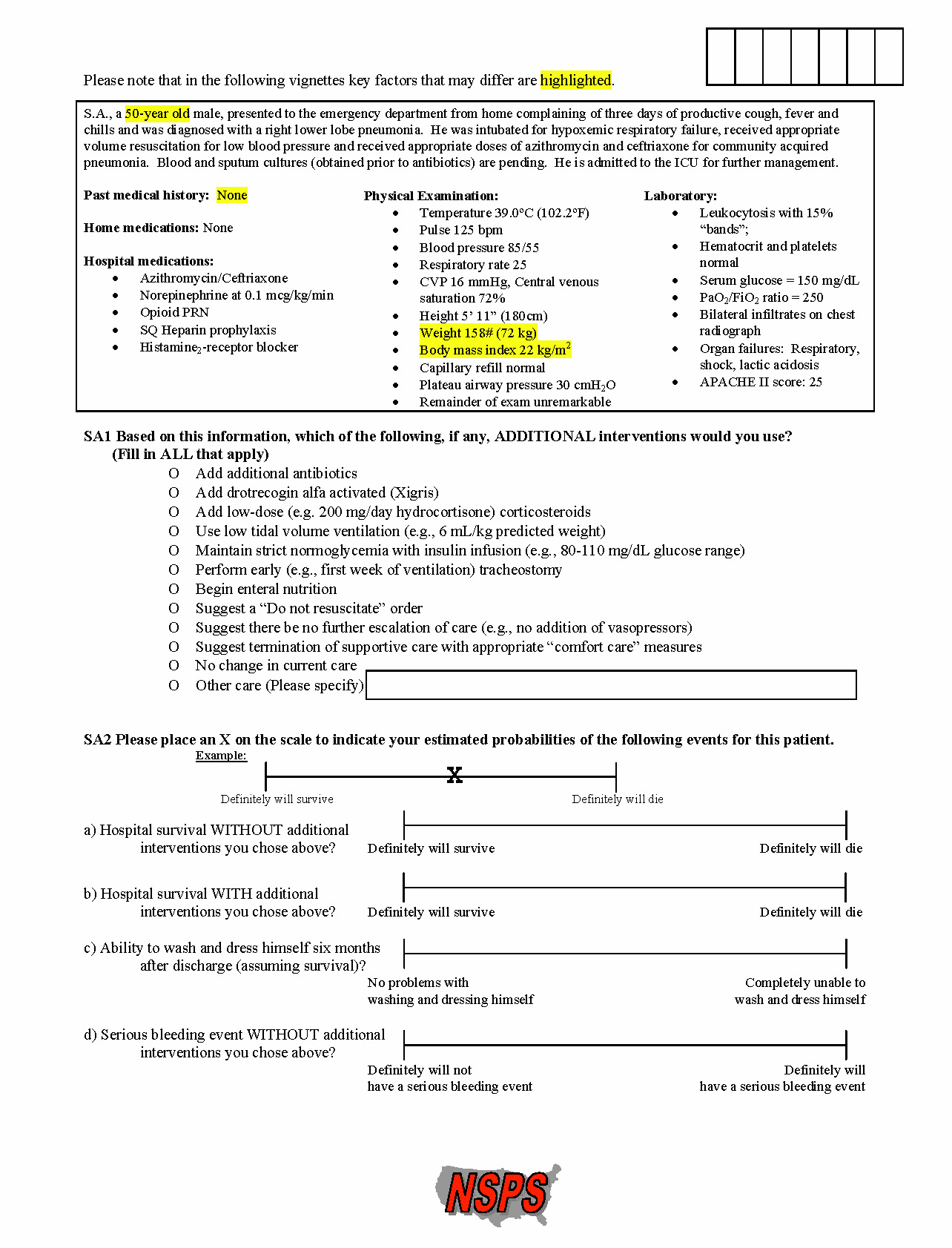

Supplement: Additional file 1 — Additional data file 1 is a JPG file containing a figure showing the 'lowest risk' vignette. The shaded areas indicate where there were variations between the vignettes (e.g. 50 years old vs. 70 years old). Each respondent received four vignettes. The first two were constant for all respondents and included this 'lowest risk' vignette and the 'highest risk' vignette (70 years old, stage IIA non-small cell lung cancer, and obese body mass index). The remaining two vignettes were randomly selected. [file cc7926-S1.jpeg]
